# Patient-Perpetrated Harassment Policies in Patient Bills of Rights and Responsibilities at US Academic Medical Centers

**DOI:** 10.1001/jamanetworkopen.2020.16267

**Published:** 2020-09-15

**Authors:** Elizabeth M. Viglianti, Lisa M. Meeks, Andrea L. Oliverio

**Affiliations:** 1 Division of Pulmonary and Critical Care Medicine, Department of Internal Medicine, University of Michigan, Ann Arbor; 2 Institute of Health Policy and Innovation, University of Michigan, Ann Arbor; 3 Department of Family Medicine, University of Michigan, Ann Arbor; 4 Division of Nephrology, Department of Internal Medicine, University of Michigan, Ann Arbor

## Abstract

This cross-sectional study examines the degree to which patient bills of rights and responsibilities from 50 academic hospitals in the US communicate a zero-tolerance policy against patient-perpetrated sexual harassment toward health care professionals.

## Introduction

The National Academies of Science, Engineering, and Medicine (NASEM) report on sexual harassment recommends that hospitals maintain a clearly written patient bill of rights and responsibilities communicating a zero-tolerance policy for sexual harassment toward health care professionals.^1^ Compliance with this recommendation among US academic medical centers is unknown; however, a previous study^2^ suggests that the top US academic medical centers lack policies for patient-perpetrated sexual harassment and guidance for trainee response. Therefore, we sought to examine the degree to which hospitals affiliated with the Association of American Medical Colleges (AAMC) complied with NASEM recommendations for addressing patient-perpetrated sexual harassment through a patient bill of rights and responsibilities and the degree to which language about patients’ rights mirrors the language about patients’ responsibilities.

## Methods

Between February and October 2019, we conducted a cross-sectional evaluation of patient bills of rights and responsibilities in 50 hospitals, randomly selected from 418 AAMC-affiliated hospitals using a random-number generator function in Stata, version 15.1 (StataCorp LLC). Two of us (E.M.V., A.L.O.) independently reviewed and coded these documents for (1) a specific statement about patients’ responsibilities to refrain from harassment and sexual harassment and their right to receive care free of harassment and (2) the tone of the language communicating this expectation. The University of Michigan institutional review board deemed this study exempt because it did not meet the definition of human subjects research. The study followed the Strengthening the Reporting of Observational Studies in Epidemiology (STROBE) reporting guideline.

Condemnation of patient-perpetrated harassment was evaluated in the patient bill of responsibilities. If the document used the words *harassment*, *abuse*, or *discrimination* to specifically condemn behaviors (eg, “harassment will not be tolerated”), it was coded as *specific*. Patient bills of responsibilities that did not use these words but that used positive behaviors to describe expectations (eg, “be considerate”) were coded as *suggestive*. If neither approach was taken, this language was coded as *absent*. The same framework was used when examining patient bills of rights for expectations regarding harassment. This coding is presented in counts (percentages).

## Results

All 50 hospitals maintained a publicly available patient bill of rights. Of these bills of rights, 47 (94%) were coded as *specific* (19 used the word *discrimination,* and 28 used *harassment* or *abuse*) because they clearly stated that the patient has the right to a discrimination-free experience (Table). Only 11 (22%) specifically addressed sexual abuse or harassment (Figure, A and Table).

**Table.** Examples of Language Used in Patient Bills of Rights and Responsibilities

Language type^a^	Patient bill of rights	Patient bill of responsibilities
Specific	“We will protect you from all forms of abuse, neglect, or harassment.” “[You have the right] to be free from all forms of abuse or harassment.” “You have the right to appropriate medical and nursing services without discrimination based upon age, race, ethnicity, religion, culture, language, physical or mental disability, socioeconomic status, sex, sexual orientation, and gender identity or expression.” “You have the right to receive care in a safe environment and be free from neglect, exploitation, and verbal, mental, physical, and sexual abuse.”	“Threats, violence, or harassment of other patients and hospital staff will not be tolerated.”
Suggestive	“[You have the right] to be treated with dignity and respect.”	“Refrain from foul, threatening, or inappropriate language.” “[You have the responsibility] to respect the dignity and right to privacy of other patients and your health care team.” “[You have the responsibility] to be considerate and cooperative.” “[You have the responsibility] to respect the rights and property of others.” “[You have the responsibility] to be considerate of the rights of other patients and facility personnel.”

^a^ Documents with specific language used the words *harassment*, *abuse*, or *discrimination*. Those with suggestive language mentioned behaviors such as *be considerate.*

**Figure.**
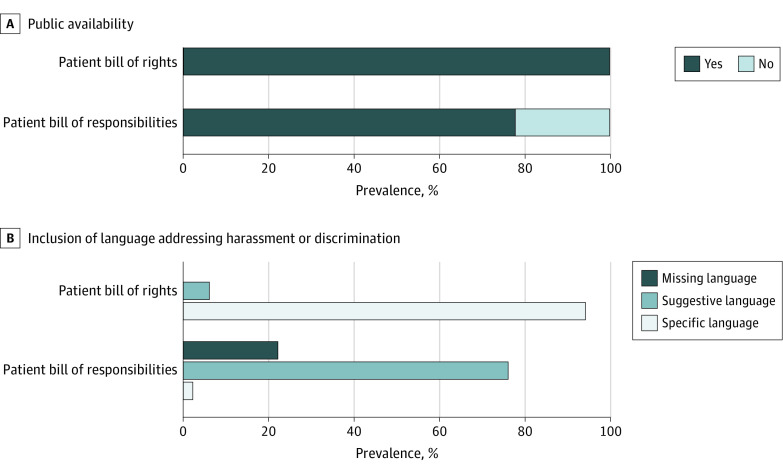
Characteristics of Patient Bills of Rights and Responsibilities From 50 Academic Hospitals Specific language uses the words *harassment*, *abuse*, or *discrimination*. Suggestive language states positive behaviors such as being *kind* or *considerate*.

Regarding patient bills of responsibilities, 39 (78%) of the hospitals maintained a publicly available statement. Of the 39 statements, 1 (3%) was coded as *specific* (it used the word *harassment*) (Table), whereas none contained language against patient-perpetrated sexual harassment or abuse (Figure, B). Language coded as *suggestive* was used in 38 (97%) of the patient responsibility statements, including that patients were to be *considerate* of hospital staff (Table).

## Discussion

In this representative sample of AAMC-affiliated academic hospitals, nearly all of the hospitals (94%) specifically delineated patients’ rights to receive care free of harassment; however, the same emphasis on zero tolerance of harassment toward health care workers was rarely included in the patients’ responsibilities. Furthermore, the tone of the language used to describe patient responsibilities was suggestive rather than specific as in the patients’ rights and was in contrast to NASEM recommendations.

One limitation of this analysis is that the investigators were not blinded to the hospitals. Another limitation is a lack of generalizability to hospitals outside the AAMC. However, patient-perpetrated harassment is commonly experienced by trainees and is associated with isolation and burnout.^3,4,5,6^ Thus, this group of hospitals warrants specific attention.

Further investigation is needed to understand how patient bills of rights and responsibilities are disseminated, viewed, and interpreted among patients. Combating patient-perpetrated harassment may require hospitals to directly address patients by maintaining clearly written expectations with congruent language in the patient bill of rights and responsibilities.
